# On the Electrical Resistance Relaxation of 3D-Anisotropic Carbon-Fiber-Filled Polymer Composites Subjected to External Electric Fields

**DOI:** 10.3390/membranes11060412

**Published:** 2021-05-30

**Authors:** Pei Huang, Yingze Cao, Zhidong Xia, Pengfei Wang, Shaosong Chen

**Affiliations:** 1 School of Energy and Power Engineering, Nanjing University of Science and Technology, Nanjing 210094, China; huangpei@emails.bjut.edu.cn; 2 Qian Xuesen Laboratory of Space Technology, China Academy of Space Technology, Beijing 100094, China; caoyingze@qxslab.cn; 3 Faculty of Materials and Manufacturing, Beijing University of Technology, Beijing 100124, China

**Keywords:** resistance relaxation, electric field, conductive polymer composite, piezoresistivity

## Abstract

Flexible composites as sensors are applied under a small voltage, but the effect of the external electrical field on the resistance is always ignored and unexplored by current research. Herein, we investigate the electrical resistance relaxation of anisotropic composites when they are subjected to an external electric field. The anisotropic composites were 3D-printed based on carbon-fiber-filled silicon rubber. Constant DC voltages were applied to the composites, and the output electrical current increased with time, namely the electrical resistance relax with time. The deflection and migration of carbon fibers are dominantly responsible for the resistance relaxation, and the angle’s evolution of a carbon fiber, under the application and removal of the electrical field, was well observed. The other factor hindering the resistance relaxation is the increased temperature originating from the Joule heating effect. This work provides a new understanding in the working duration and the static characteristics of flexible composites.

## 1. Introduction

Flexible conductive composites have been widely fabricated as various sensors due to their response of electrical signals to various loadings [1,2]. Various electrical signals have been devoted to characterizing the sensing properties, including electrical resistance [3], capacitance [4], current [5,6], voltage [7] and so on.

The work duration and static characteristics of flexible sensors are of great significant to evaluate the sensing stability and reliability, especially referring to the piezoresistive sensors [8]. Constant input of compressive stress (static characteristics) is desirable to have a stable feedback of electrical signal. However, the flexible composites always suffer from resistance creep, relax or hysteresis when compressed [9,10,11], which is highly related to the viscoelasticity of the flexible matrix. Having been extensively studied, the resistance creep or relaxation under compressive loadings is always analyzed with the effect of loading stress; however, the influence of external electric field is highly ignored and unexplored. Multimeter or LCR bridge in a testing circuit are widely used to record the sensing electrical signals, whose inner voltage are simultaneously applied on the tested samples.

Recently, our group 3d-printed an anisotropic composite based on carbon-fiber-filled conductive silicon rubber [12], and the printing process is depicted in Figure S1. The oriented carbon fibers resulted in the electrical anisotropy of the composites, and better conductivity was gained in the orientation direction (Figures S2 and S3). The printed polymer composites were further investigated in this paper.

Occasionally and interestingly, we found the characterized inherent resistance (>100 Ω) of various conductive polymer composites to be unstable when tested with a multimeter, and it was further proved in our printed composites (Figure S4), in which the resistance of printed composites decreased by 14% after 10 min test with a multimeter. We noticed that the tested specimens were free of stress load, which reminded us of the ignored factor of external electric field originated from the test multimeter (0.56 V).

Herein, the behavior of the printed composites under the external electric field is studied. The roles of Joule heating effect and the migration of fibers played on the resistance relaxation were experimentally characterized, in-situ observed and schematically revealed. The effect of the electrical field on the piezoresistivity was finally tested. All the experimental results would do a favor in better understanding on the working duration of conductive composites and sensors.

## 2. Materials and Methods

### 2.1. Materials and preparation

The printing process was based on an extrusion device cooperated with a desktop printer and it was controlled by a computer. The printed composites were then pre-cured at 150 °C for 5 min and post-cured at 170 °C for 10 min. The polymer matrix material (methyl vinyl rubber) and vulcanizing agent were obtained from Blue-star Silicone Co. Ltd. Shanghai, China. The fillers of short carbon fibers purchased from Zhongli New Material Technology Co. Ltd. Cangzhou, China, whose nominal length was180 μm and diameter was 8 μm. The 3D printing device for the tested samples is shown in Figure S1.

### 2.2. Characterization

The method to characterize the resistance relaxation behavior of the printed composites under the external electric field is depicted in Figure S5. Polymer composites as sensors are generally utilized under a small voltage, and the constant DC voltages of 3, 5 and 10 V were therefore adopted on the composites. The changes of electrical current values of the printed composites within 12 h were recorded with a multimeter, and the interval time was set as 20 s. The tested specimens, with a dimension of 50 × 10 × 1 mm, were characterized in the *∥* and *⊥* direction, respectively. *∥* and *⊥* represent test parallel and perpendicular to the orientation direction of the anisotropic composites, respectively.

The piezoresistivity test of the printed composites is depicted in Figure S6. One sample in *∥* direction and one sample in *⊥* direction, with the dimension of 50 mm × 10 mm ×1 mm, was respectively inserted into the test fixture. Pressure was loaded on the test fixture by the counterweights, and the pressure interval was 20 kPa. Multimeter (Victor 86E) was connected to both ends of the printed composite to record the resistances.

## 3. Results

**Figure 1:**
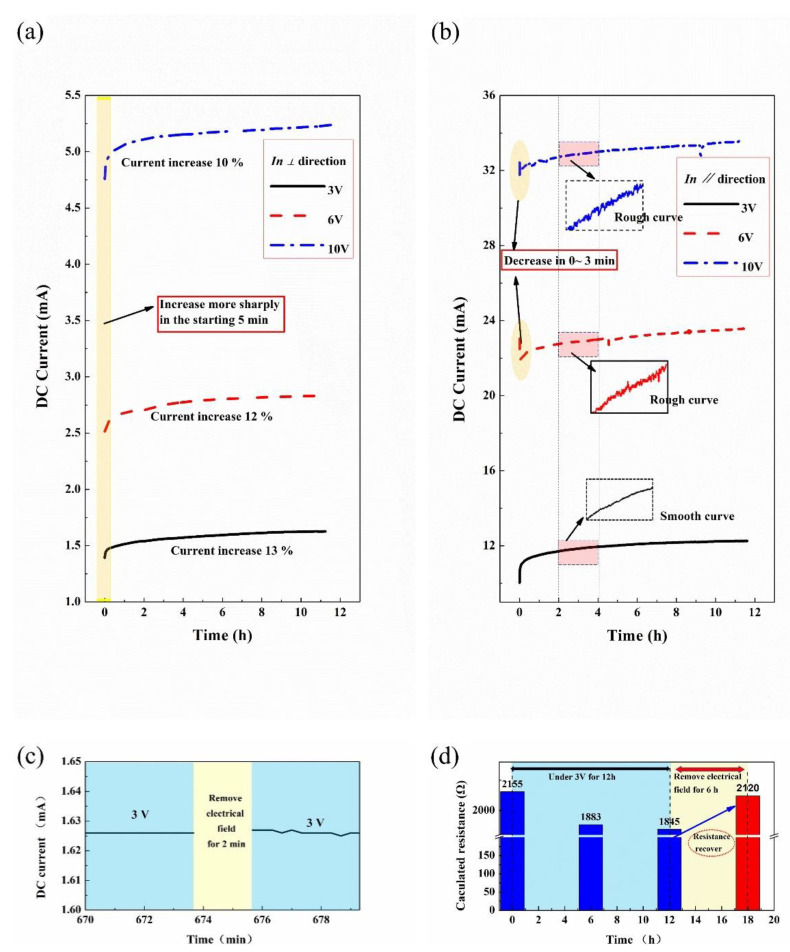
Electrical current changes (resistance relaxation) of printed composites within 12 h under DC voltages. (**a**) In *⊥* direction. (**b**) In *∥* direction. (**c**) Under 3 V for about 673 min, and then the electrical field removed for 2 min, and then the electrical field applied again. Resistance stayed constant with the state before the removal of electrical field. (**d**) Resistance recovers to its original state after the removal of electrical field for 6 h.

The resistance relaxation of the printed composites under the external electric field is studied, and the experimental results in *⊥* direction are displayed in Figure 1a. It can be seen that the electrical currents of the composites gradually increase within 12 h under 3~10 V, in which the values increase even more sharply in the 0~5 min and turn out to be stable after several hours. By electrifying the composites under 3, 6 and 10 V, the resulted current values increase by (*dI*) 13%, 12% and 10%, respectively, which indicate the resistance relaxation (*dR*) of 13%, 12% and 10%, respectively, according to Formula (1).

(1) $$dR=\frac{U}{dI}$$

The results demonstrate the stable resistance value is about 85~90% of the starting original test value. Our finding proves the thoughtless of related researched on the resistance creep in piezoresistive composites. Comprehensive consideration, of the combined effect of compressive load and electric field, is greatly helpful for recognizing and redefining the piezoresistive effect. What is emphasized is the current value is small (<6 mA) in the conductive path of *⊥* direction.

The experimental curves are a bit different in the *∥* direction, as shown in Figure 1b. The overall trend of the curves in the *∥* direction is similar to that in *⊥* direction: namely, the electrical resistance relaxes with time under the external electric fields of 3~10 V. The curves slightly changed when the applied voltage further increased to 6 and 10 V, with the resulted electrical currents over 20 mA. To the contrary, the electrical current decreases (resistance creep) rapidly in the 0~3 min instead of the resistance relaxation in a low resulted current, which is highlighted with a yellow ellipse in Figure 1b. Moreover, the experimental curves tend to be rough at the voltages of 6 and 10 V rather than be smooth at 3 V, which is highlighted with red rectangles in Figure 1b. The rough curves can be ascribed to the unstable state of fibers in the printed composites.

To judge whether the resistance relaxation is reversible, a printed sample was applied under a DC voltage of 3 V for about 12 h, and then the electric field was removed for a short time and a long time, respectively, and the electric field was applied again. The demonstrations are described in Figure 1c,d. According to Figure 1c, the electrical current could stay constant with the state before the removal of electrical field for 2 min, verifying that resistance relaxatation could stay stable in a short time (<2 min). According to Figure 1d, the resistance (*R = U/I*) is calculated, with resistance relaxation from 2155 to 1845 Ω after the 12 h of applied voltage, and it recovers to 2120 Ω after the removal of electrical field for 6 h, which demonstrates the reversibility of the resistance relaxation after a long time (>6 h) removal of applied electrical field and further refers to the reversible transformation of the conductive pathway in the printed composites after the removal of electrical field.

Two factors may weigh for the unusual electrical resistance relaxation as well as the rough curves, i.e., (1) according to the Joule heating effect, the temperature of composites increased during power-on, resulting in the positive temperature coefficient effect of the electrical resistance, and the resistance further increased. (2) The filler of carbon fibers may deflect and migrate under the external electric field, bringing about the rearrangements of conductive paths of the composites, and the resistances are therefore influenced.

The temperatures changes are characterized with an infrared thermal image (FLIR-E6390). Joule’s law expresses the relationship of heat generated by the applied voltage (Formula (2)), and the temperature of the composites increases when the generated heat (Q) is higher than the lost heat.

(2) $$Q=\frac{U^{2}}{R}t$$

**Figure 2:**
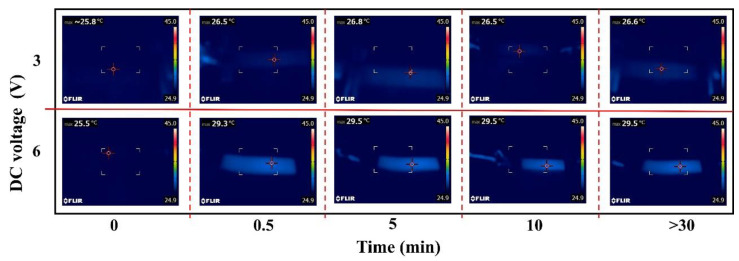
Temperatures changes under the applied voltage of 3 and 6 V, tested in *∥* direction.

The high resistance (≈2 kΩ) in *⊥* direction substantially decreases the generated heat, and the corresponding temperature stays unchanged. The tests of the composites in *∥* direction under 3 and 6 V are therefore depicted in Figure 2. It can be concluded that the temperatures increased within 0~0.5 min and remained stable (0.5~12 h) though the voltages were still applied. The increased temperatures increase the electrical resistance, and the correlation is described in Figure S7 and our previous work [13]. The temperatures increase about 1 °C and 4 °C at the voltage of 3 and 6 V, respectively. The applied voltage of 3 V barely has any influence on the resistance of composites as seen in the relatively stable temperatures, whereas the applied voltage of 6 V may result in about 4% increase of the resistance value (in *∥* direction) for the temperature increase from 25 to 29 °C (Figure S7). The variety of temperatures increase, at 3 and 6 V, can be further used to explain the different trends in the Figure 1b at the 0~3 min.

**Figure 3:**
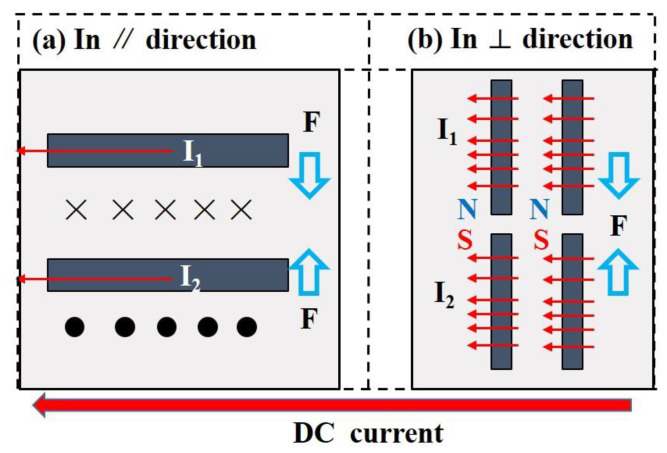
Schematic diagrams of the interaction between carbon fibers under the DC current. (**a**) In *∥* direction. (**b**) In *⊥* direction. Black rectangles denote carbon fibers.

The factor of Joule heating effect increases the resistance and promotes the resistance creep, which is contrary to the behaved resistance relaxation in Figure 1. Therefore, we consider that the factor of the deflection of carbon fibers is dominant in the resistance relaxation. We hereby propose the schematic diagrams of the interaction between carbon fibers under the external electric field, as shown in Figure 3. The printed composites possess abundant conductive pathways, and the magnetic field is induced when the electric field is adopted. The induced magnetic field further functions on the conductive carbon fibers. In *∥* direction (Figure 3a), the two carbon fibers can be viewed as two wires, with the current having the same direction, and the interaction force can be analyzed with Formula (3).

(3) $$F=\frac{uI_{1}I_{2}L}{2\pi d}\;$$

where *u* denotes magnetic permeability; *I*_1_ and *I*_2_ are the current in two carbon fibers; and *L* and *d* represent the constant parameters of the carbon fibers. The inter-force between the two fibers indicates that the carbon fibers tend to get close to each other, as well as deflect to the direction of electrical current. As for the characterization in *⊥* direction, the carbon fibers can be regarded as spiral coils with opposite magnetic poles, also resulting in the approach of fibers.

The approached fibers form more conductive pathways that further dominate the electrical relaxation described in Figure 1, and the curves turn to be stable after several hours owing to the stable state of the migrated fibers. The roughness of curves in Figure 1b can also be ascribed to the higher inter-force originated from the higher applied currents.

**Figure 4:**
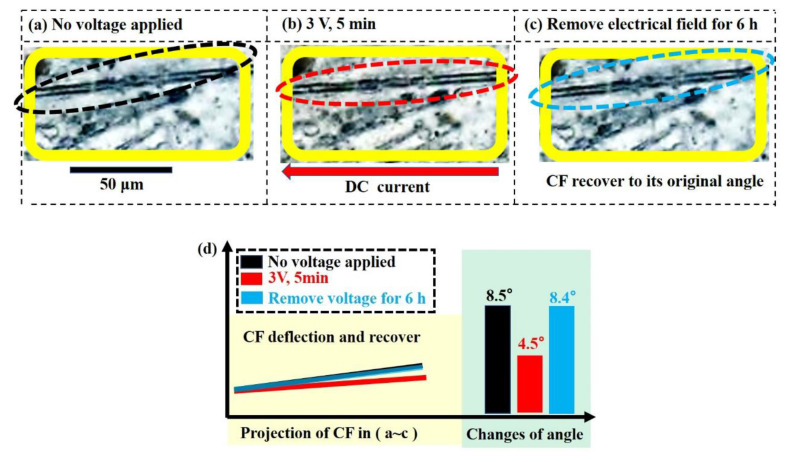
Micromorphology of a fiber: (**a**) no voltage applied, (**b**) under 3 V for 5 min and (**c**) removed electrical field for 6 h. (**d**) Projection of the carbon fiber in (**a**–**c**) and the changes of the corresponding angle.

To verify our explanation about the interaction between carbon fibers, the deflection of carbon fibers under electric field of 3 V and 5 min are observed in-situ, as shown in Figure 4a,b. The removal of applied electrical field on the microstructure was also characterized as depicted in Figure 4c, and the angle’s evolution of one marked carbon fiber was summarized in Figure 4d by the projection of the marked carbon fiber from Figure 4a–c. Though it is hard to distinguish the tiny displacement of fibers, the deflection of carbon fiber is still well demonstrated by the fiber in the elliptical in Figure 4a–d. The characterized fiber tends to rotate to the direction of the applied electrical current (8.5° → 4.5°), further resulting in the resistance relaxation. The deflection of the characterized is reversible after the removal of electrical field for 6 h (4.5° → 8.4°), which is very consistent with the resistance recover and reversibility process plotted in Figure 4d. Before our observation on the fiber rotation in the solidified composites when electrified, there was only the demonstration in the uncured polymer under a high electrical field [14].

**Figure 5:**
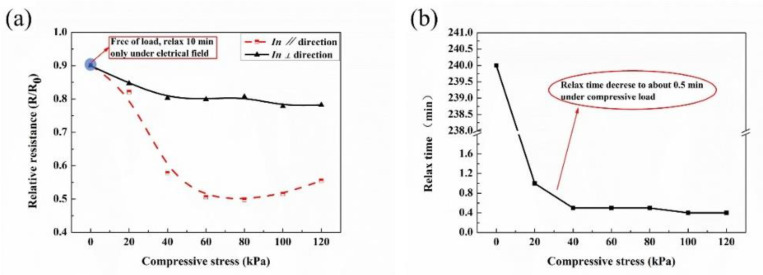
Static piezoresistivity of the printed composites under compressive stress and a test voltage of 0.56 V. (**a**) Resistance response to compressive load, and (**b**) relax time of the composites, referring to the time of the resistance reaches to a stable value.

The aforementioned experimental results inspired us to verify the role of electrical field on the piezoresistivity; the result is demonstrated in Figure 5, and the test method is depicted in Figure S6. The static resistance of printed composites function with compressive stress is plotted in Figure 5a, where the *R*_0_ denoted the initial value when the composites connected into test circuit, and the stable resistance values (*R*) were recorded. As shown in the blue circle in Figure 5a, in which resistance relaxes only under electrical field (free of load) for 10 min, the corresponding relative resistance decrease to about 0.9. The printed composites exhibited negative compressive resistance effects in both directions, and they were more compressive-sensitive in *∥* direction. The compressive load together with the electrical field are responsible for the downward trend of Figure 5a, and the electrical field plays a more non-ignorable role, especially in *⊥* direction with a higher relative resistance in *⊥* direction. The other need to be highlighted is described in Figure 5b, and the time of the resistance relaxation to a stable value greatly reduced from several hours to about 0.5 min, when the printed composites were compressed.

## 4. Conclusions

In conclusion, this paper stresses the ignored effect of the electric field on the resistance relaxation/creep of flexible composites. The resistance relaxation of the printed composites under the external electric field (3–10 V) is well revealed. Resistance relaxes sharply in the 0~5 min and becomes stable later in the *⊥* direction, while slightly different in *∥* direction. Two factors’, including the Joule heating effect and interaction between fibers, weight for the resistance relaxation are experimentally discussed and verified. Our work will be beneficial for the better understanding on the working duration and static characterizations of flexible composites.

## Data Availability

The data that support the findings of this study are available within the article (and its supplementary material).

## Supplementary Materials

The following are available online at https://www.mdpi.com/article/10.3390/membranes11060412/s1, Figure S1: 3D printing device for the tested samples. The printing process was based on an extrusion device cooperated with a desktop printer and it was controlled by a computer. The printed composites were then pre-cured at 150 °C for 5 min and post-cured at 170 °C for 10 min. The polymer matrix material (methyl vinyl rubber) and vulcanizing agent were obtained from Blue-star Silicone Co. Ltd. Shanghai, China. The fillers of short carbon fibers purchased from Zhongli New Material Technology Co. Ltd. Cangzhou, China, whose nominal length was180 μm and diameter was 8 μm; Figure S2: Carbon fibers oriented along the printing direction in the as-printed composites; Figure S3: Anisotropy of volume resistivity of the as-printed composite in different test Directions [13]; Figure S4: Unstable inherent resistance characterization of a printed composite test with a multimeter (0.56 V). The relative resistance decreases 0.14 after 10 min of test, and the R0 is about 250 Ω in the ∥ direction; Figure S5: Experimental device for the test of resistance relaxation under external electrical fields. The composite was printed with its dimension of 50 mm × 10 mm × 1 mm. The voltage source (JC1733S) supplied DC voltages of 3 V, 5 V and 10 V. The time interval of the multimeter was set as 20 s and the data was recorded with computer. The printed composite, under the voltage of 3 V, was also in-situ observed under a metallographic microscope (BX51M); Figure S6: Schematic illustration for the test of piezoresistivity. In the compressing test, one sample in ∥ direction and one sample in ⊥ direction, with the dimension of 50 mm × 10 mm × 1 mm, was respectively inserted into the test fixture. Pressure was loaded on the test fixture by the counterweights, and the pressure interval was 20 kPa. Multimeter (Victor 86E) was connected to both ends of the printed composite to record the resistances; Figure S7: Electrical resistance creep with the temperature increases from 25 °C to 40 °C.
