# Supplementary material for: On the Electrical Resistance Relaxation of 3D-Anisotropic Carbon-Fiber-Filled Polymer Composites Subjected to External Electric Fields
Source: Membranes (Basel). 2021 May 30;11(6):412. doi: 10.3390/membranes11060412 (PMC8228886; doi:10.3390/membranes11060412)
Supplement: Supplementary file 1 [file membranes-11-00412-s001.zip › membranes-1168889-SI.pdf]

membranes

Supporting Information

On the Electrical Resistance Relaxation of 3D-Anisotropic Carbon-Fiber-Filled Polymer Composites Subjected to External Electric Fields

Pei Huang [1,2], Yingze Cao [2], Zhidong Xia [3,*], Pengfei Wang [2,*] and Shaosong Chen [1,*]

1 School of Energy and Power Engineering, Nanjing University of Science and Technology, Nanjing 210094, China; huangpei@emails.bjut.edu.cn
2 Qian Xuesen Laboratory of Space Technology, China Academy of Space Technology, Beijing 100094, China; caoyingze@qxslab.cn
3 Faculty of Materials and Manufacturing, Beijing University of Technology, Beijing 100124, China
* Correspondence: xiazhd@bjut.edu.cn (Z.X.); wangpengfei@qxslab.cn (P.W.); chenss805@163.com (S.C.)

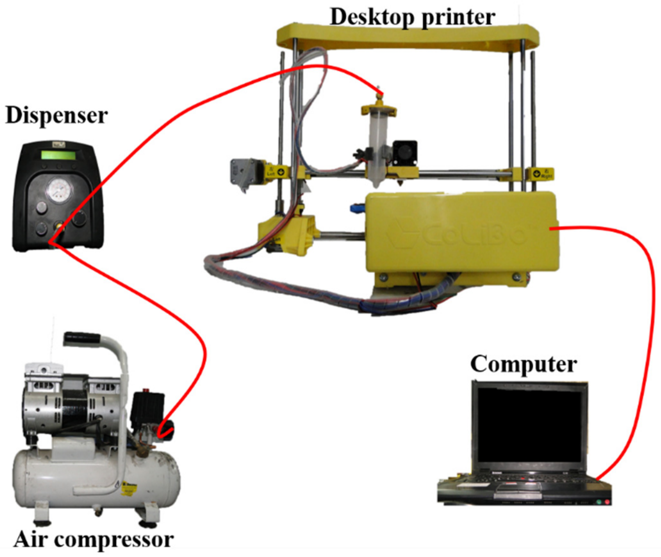

Figure S1. 3D printing device for the tested samples. The printing process was based on an extrusion device cooperated with a desktop printer and it was controlled by a computer. The printed composites were then pre-cured at 150 °C for 5 min and post-cured at 170 °C for 10 min. The polymer matrix material (methyl vinyl rubber) and vulcanizing agent were obtained from Blue-star Silicone Co. Ltd. Shanghai, China. The fillers of short carbon fibers purchased from Zhongli New Material Technology Co. Ltd. Cangzhou, China, whose nominal length was180 μm and diameter was 8 μm.

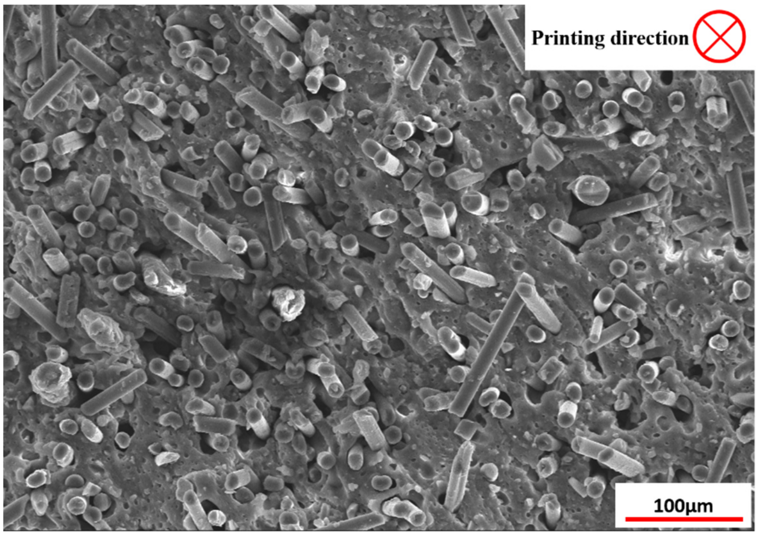

Figure S2. Carbon fibers oriented along the printing direction in the as-printed composites.

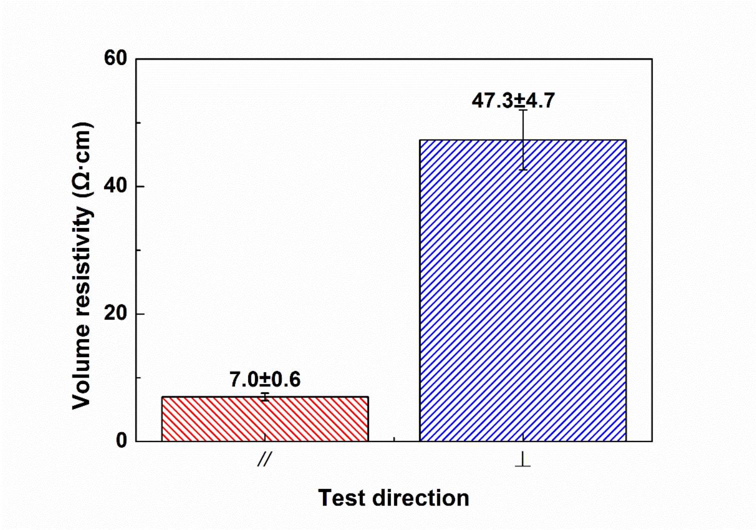

Figure S3. Anisotropy of volume resistivity of the as-printed composite in different test. Directions [13]

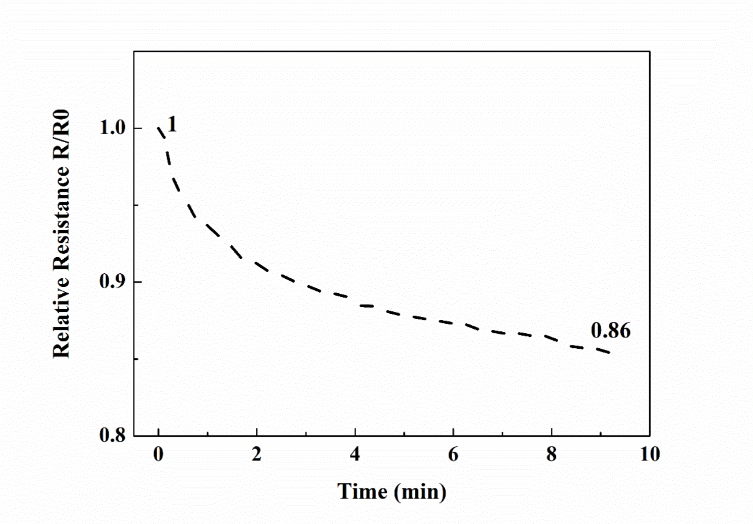

Figure S4. Unstable inherent resistance characterization of a printed composite test with a multimeter (0.56 V). The relative resistance decreases 0.14 after 10 min of test, and the R_0 is about 250 Ω in the // direction.

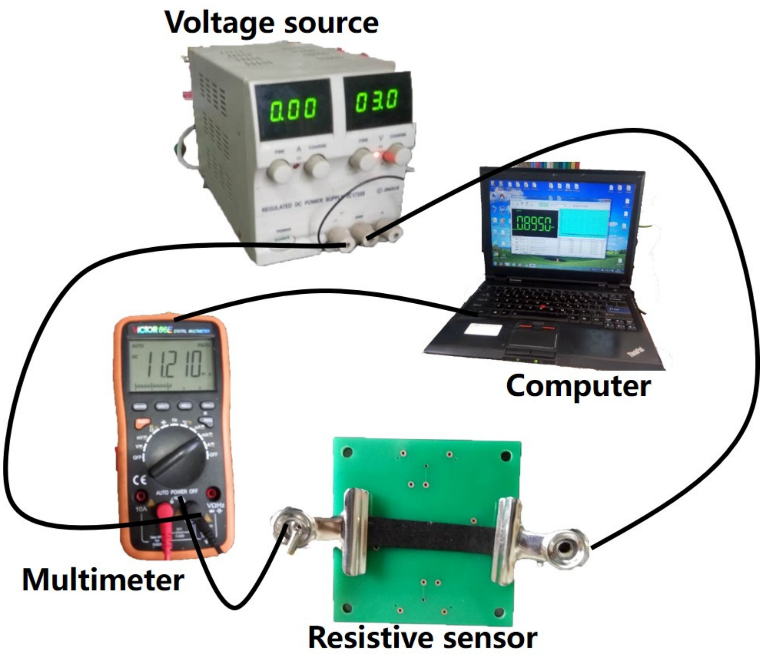

Figure S5. Experimental device for the test of resistance relaxation under external electrical fields. The composite was printed with its dimension of 50 mm× 10 mm ×1 mm. The voltage source (JC1733S) supplied DC voltages of 3 V, 5 V and 10 V. The time interval of the multimeter was set as 20s and the data was recorded with computer. The printed composite, under the voltage of 3 V, was also in-situ observed under a metallographic microscope (BX51M).

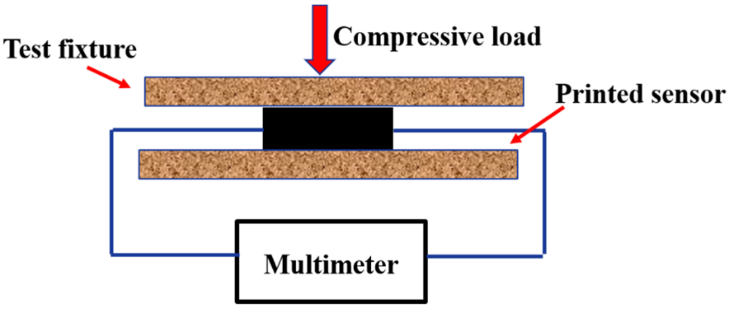

Figure S6. Schematic illustration for the test of piezoresistivity. In the compressing test, one sample in // direction and one sample in ⊥ direction, with the dimension of 50 mm × 10 mm ×1 mm, was respectively inserted into the test fixture. Pressure was loaded on the test fixture by the counterweights, and the pressure interval was 20 kPa. Multimeter (Victor 86E) was connected to both ends of the printed composite to record the resistances.

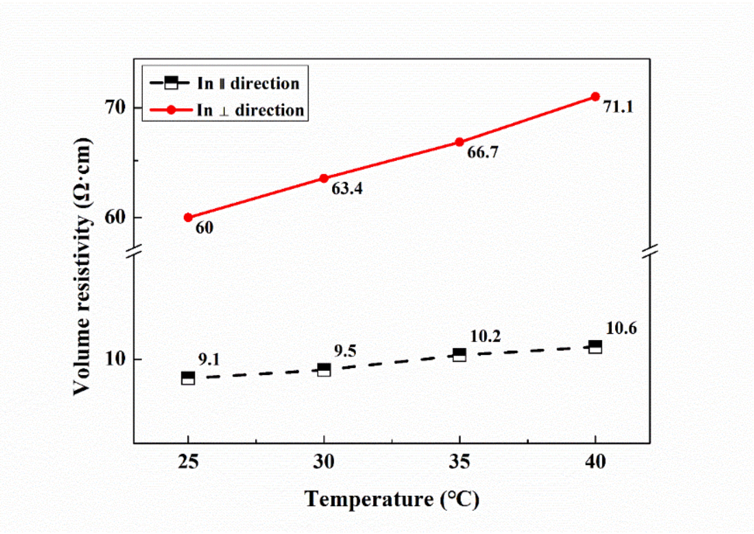

Figure S7. Electrical resistance creep with the temperature increases from 25 °C to 40 °C.
